# Spontaneous rupture of an undifferentiated carcinoma with osteoclast-like giant cells of the pancreas presenting as intra-abdominal bleeding: a case report

**DOI:** 10.1186/s40792-022-01437-2

**Published:** 2022-04-29

**Authors:** Hideo Tomihara, Kazuhiko Hashimoto, Hajime Ishikawa, Daisuke Terashita, Atsushi Gakuhara, Shuichi Fukuda, Katsuya Ohta, Kotaro Kitani, Jin-ichi Hida, Tomoko Wakasa, Yutaka Kimura

**Affiliations:** 1 Department of Surgery, Kindai University Nara Hospital, 1248-1 Otoda-cho, Ikoma, Nara 630-0293 Japan; 2 Department of Pathology and Laboratory, Kindai University Nara Hospital, 1248-1 Otoda-cho, Ikoma, Nara 630-0293 Japan

**Keywords:** Undifferentiated carcinoma, Osteoclast-like giant cell, Spontaneous rupture

## Abstract

**Background:**

Undifferentiated carcinoma is a very rare histologic subtype, representing only 0.8% to 5.7% of all pancreatic exocrine neoplasms. Additionally, spontaneous abdominal hemorrhage is a particularly rare, life-threatening cause.

**Case presentation:**

A 68-year-old man was taken by ambulance to our hospital because of sudden-onset abdominal pain. Contrast-enhanced abdominal computed tomography revealed a huge mass measuring 99 × 70 mm in the pancreatic tail with enhanced rim staining in the peripheral area. Imaging also showed extravasation and fluid collection beside the tumor. Hence, spontaneous rupture of the pancreatic tumor and intra-abdominal bleeding were diagnosed. Emergency laparotomy was performed because of acute abdominal pain with peritoneal signs. With an intraoperative diagnosis of rupture of the pancreatic tumor, distal pancreatectomy was successfully performed. Histologically, hematoxylin and eosin staining showed round to spindle-shaped, highly pleomorphic mononuclear cells and multinucleated giant cells as well as a component of ductal adenocarcinoma. Immunohistochemical staining showed that the tumor cells were negative for AE1/AE3, whereas the non-neoplastic osteoclast-like giant cells were positive for CD68. Taken together, these results led to a diagnosis of undifferentiated carcinoma with osteoclast-like giant cells. The patient’s postoperative course was uneventful.

**Conclusion:**

We experienced an extremely rare case of spontaneous rupture of an undifferentiated carcinoma with osteoclast-like giant cells presenting as intra-abdominal bleeding. Obtaining a correct preoperative diagnosis is quite difficult at the first evaluation. Undifferentiated carcinoma should be considered as a differential diagnosis in the case with spontaneous rupture of a pancreatic tumor.

**Supplementary Information:**

The online version contains supplementary material available at 10.1186/s40792-022-01437-2.

## Background

Undifferentiated carcinoma of the pancreas (UCP) is an extremely rare histologic subtype of pancreatic cancer that accounts for 0.8% to 5.7% of all pancreatic exocrine neoplasms [1–3]. UCP is well known to be associated with an aggressive malignant potential and a poorer prognosis than pancreatic ductal adenocarcinoma. UCP was first reported as pleomorphic carcinoma by Sommers and Meissner [4]. The definition of UCP is often confusing. It is because various terms have been used to describe different subtypes of UCP, including anaplastic carcinoma, pleomorphic carcinoma, pleomorphic large cell carcinoma, pleomorphic giant cell carcinoma, spindle cell carcinoma, sarcomatoid carcinoma, and carcinosarcoma. In the World Health Organization classification of tumors of the digestive system in 2010, these carcinomas were defined as undifferentiated carcinoma in which a significant component of the neoplasm does not show a definitive direction of differentiation [5]. However, undifferentiated carcinoma with osteoclast-like giant cells was discriminated as a different subtype and a distinct entity from other subtypes. Additionally, spontaneous abdominal hemorrhage is a particularly rare, life-threatening cause. We herein present our experience with an extremely rare case of spontaneous rupture of a UCP with osteoclast-like giant cells presenting as intra-abdominal bleeding.

## Case presentation

A 68-year-old man with a history of hypertension, diabetes mellitus, and myocardial infarction was taken by ambulance to our tertiary referral hospital’s emergency department because of sudden-onset left upper quadrant pain of 1-h duration accompanied by nausea and sweating. The patient had a treatment history of percutaneous coronary intervention and placement of three stents in his coronary arteries, and he was taking two kinds of anticoagulant drugs, 100 mg of aspirin and 75 mg of thienopiridines.

**Fig. 1:**
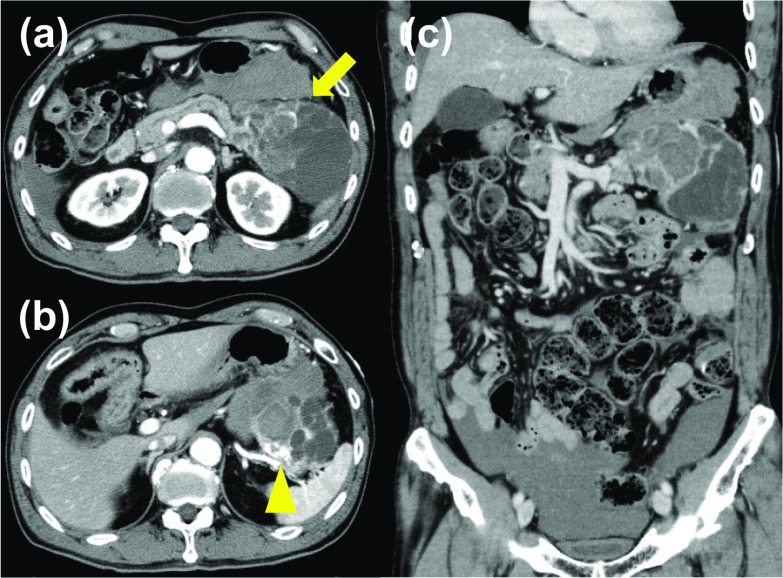
**a** Contrast-enhanced computed tomography of the abdomen showed a tumor with a maximum length of 99 mm in the pancreatic tail with enhanced rim staining in the peripheral area in the arterial phase (arrow). **b** The point of extravasation from the tumor (arrow top). **c** The coronal plane in the portal vein phase

**Fig. 2:**
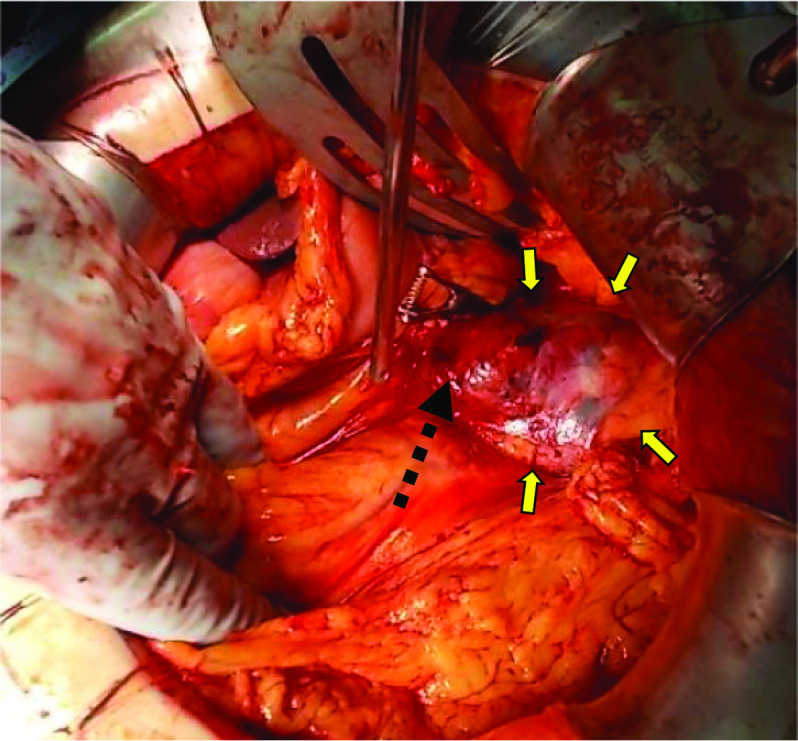
Intraoperative photograph demonstrating the cystic tumor (yellow allows). The cystic tumor in the pancreatic tail was ruptured, and active bleeding was detected at the top of the tumor (dashed line)

At the first visit, the patient’s blood pressure was 118/56 mmHg, heart rate was 82 beats/min, and body temperature was 36.8 °C. Physical examination revealed abdominal distention with tenderness in the epigastric area. Blood tests showed slight anemia (hemoglobin, 12.4 g/dL) and increased concentrations of aspartate aminotransferase (53 U/L), lactate dehydrogenase (492 U/L), d-dimers (2.9 µg/mL), and hemoglobin A1c (9.0%); his myocardial deviation enzymes were not elevated (Table 1). A cardiologist performed electrocardiogram and echocardiography to rule out acute coronary syndrome. The electrocardiogram showed a poor R-wave progression pattern in the II, III, and aVF induction leads, but echocardiography showed no apparent or significant local asynergy. There was no evidence of acute coronary syndrome. For further examination, the patient underwent abdominal contrast-enhanced computed tomography (CECT). This examination revealed a huge low-density mass measuring 99 × 70 mm in the pancreatic tail with enhanced rim staining in the peripheral area (Fig. 1a). Imaging also revealed extravasation and fluid collection beside the tumor (between the stomach and pancreas or spleen) as well as ascites under both sides of the diaphragm and in the pouch of Douglas (Fig. 1b). Hence, spontaneous rupture of a pancreatic tumor [gastrointestinal stromal tumor, mucinous cystadenoma (adenocarcinoma), or malignant lymphoma] or aneurysm were considered as differential diagnoses. Emergency laparotomy was performed. The abdominal cavity was full of gross blood and clots. Active bleeding was observed from the top of a ruptured mass in the pancreatic tail. A large tumor was found originating from the pancreatic tail (Fig. 2). There were no signs of distant metastasis, regional lymphadenopathy, or dissemination, while intra-peritoneal cytology was not examined. With an intraoperative diagnosis of spontaneous rupture of a pancreatic tumor, standard distal pancreatectomy with (regional) lymph node dissection was successfully performed.

**Table 1:** Patient’s laboratory data at admission

Laboratory test	Value	Normal range	Unit
White blood cells	6480	3300–8600	/uL
Red blood cells	426 × 10^4^	386–492 × 10^4^	/uL
Hemoglobin	12.4	11.6–14.8	g/dL
Platelets	20.3 × 10^4^	15.8–34.8 × 10^4^	/uL
Prothrombin time	109.7	80–120	%
Sodium	136	138–145	mEq/L
Potassium	5.8	3.6–4.8	mEq/L
Chloride	103	101–108	mEq/L
Total protein	6.8	6.6–8.1	g/dL
Albumin	4.0	4.1–5.1	g/dL
Total bilirubin	0.6	0.4–1.5	mg/dL
Aspartate aminotransferase	53	13–30	/uL
Alanine aminotransferase	20	7–23	/uL
Alkaline phosphatase	209	106–322	/uL
Gamma-glutamyl transpeptidase	24	9–32	/uL
Lactate dehydrogenase	492	124–222	/uL
Cholinesterase	289	201–421	/uL
Total cholesterol	133	142–220	mg/dL
Triglyceride	52	30–150	mg/dL
Blood urea nitrogen	22.9	8–20	mg/dL
Creatinine	0.9	0.46–0.79	mg/dL
Hepatitis B surface antigen	Negative	Negative	
Hepatitis C virus antibody	Negative	Negative	
Alfa-fetoprotein	2.6	0–10	ng/mL
Carcinoembryonic antigen	2.4	0–5	ng/mL
Carbohydrate antigen 19–9	7.6	0–37	U/mL
Protein induced by vitamin K absence or antagonist-II	27	0–39	mAU/mL
Hemoglobin A1c	9.0	4.6–6.2	%

**Fig. 3:**
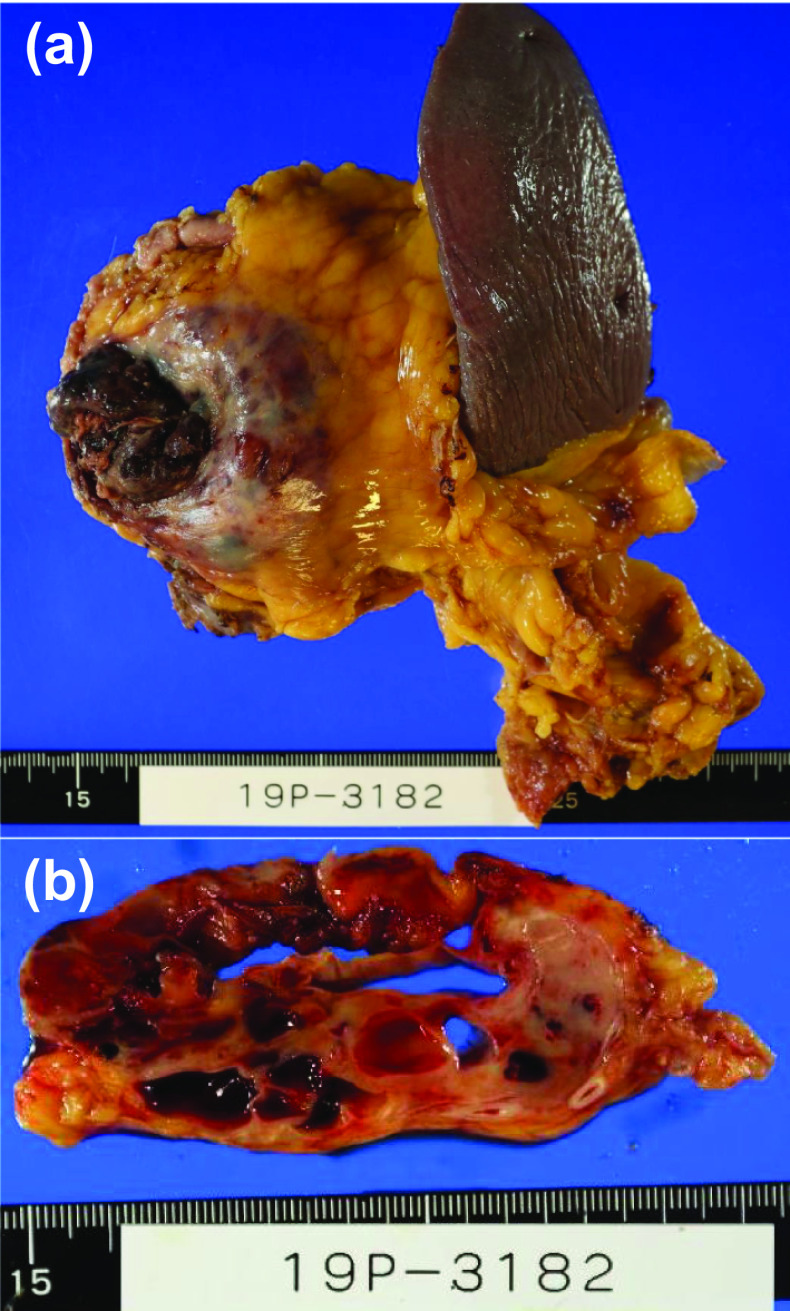
Photographs of the surgical specimen. **a** Macroscopically, the tumor was multilocular-cystic and well-defined; hemorrhage was present, and the tumor had a septum. **b** The divided surface of the tumor (arrows)

**Fig. 4:**
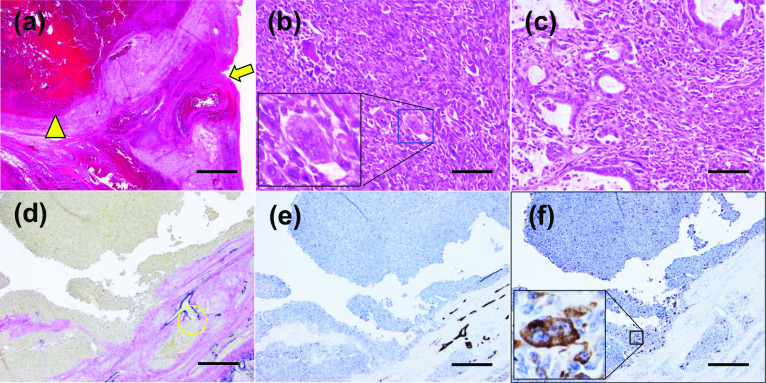
Histopathological findings of the resected specimen. **a** Hematoxylin and eosin, ×40. Scale bar = 500 µm. The site of the rupture (arrow). Intra-tumoral hemorrhage (arrowhead). **b** Hematoxylin and eosin, ×200. Non-neoplastic osteoclast-like giant cells were present (square). Scale bar = 200 µm. **c** Hematoxylin and eosin, ×200. Round to spindle-shaped, highly pleomorphic mononuclear cells as well as a component of ductal adenocarcinoma were seen. Scale bar = 200 µm. **d** Elastic fiber staining, ×40. Scale bar = 500 µm. Extensive tumor infiltration into the splenic vein was present (yellow circle). **e** Immunohistochemical examination showed that the tumor cells were negative for cytokeratin AE1/AE3, ×40. Scale bar = 500 µm. **f** The non-neoplastic osteoclast-like giant cells were positive for CD68 (square), ×40. Scale bar = 500 µm

Macroscopic examination of the resected specimen revealed that the ruptured tumor was located in the pancreatic tail. It was approximately 80 mm in diameter, multilocular-cystic, and well-defined; hemorrhage was present, and the tumor had a septum (Fig. 3a, b). Histologically, hematoxylin and eosin staining showed rupture of the tumor distension due to an intra-tumoral hemorrhage (Fig. 4a) as well as round to spindle-shaped, highly pleomorphic mononuclear cells and multinucleated histiocytic giant cells as well as a component of ductal adenocarcinoma (Fig. 4b, c). Elastic fiber staining showed extensive tumor infiltration into the splenic vein (Fig. 4d). Immunohistochemical staining showed that the tumor cells were negative for cytokeratin AE1/AE3 (Fig. 4e), whereas the non-neoplastic osteoclast-like giant cells were positive for CD68 (Fig. 4e). There was no lymph node metastasis nor perineural invasion, while there was vascular invasion in accordance with the general rules for the study of pancreatic cancer (7th edition). Taken together, these results led to a diagnosis of undifferentiated carcinoma with osteoclast-like giant cells. The patient’s postoperative course was uneventful, while the patient chose best supportive care 3 months after surgery.

## Discussion

Although there are many potential causes of acute abdomen, spontaneous abdominal hemorrhage (defined as non-traumatic and non-iatrogenic intra-abdominal bleeding) is a particularly rare, life-threatening cause [6]. Visceral sources of spontaneous intra-abdominal bleeding have been reported and include an underlying hypervascular tumor [6], spontaneous rupture of hepatocellular adenoma (adenocarcinoma) [7], renal cell carcinoma [8], renal angiomyolipoma [9], adrenal pheochromocytoma [10], and gastrointestinal stromal cell tumor [11]. However, a pancreatic tumor is an extremely rare source of spontaneous intra-abdominal bleeding. A review of the literature revealed only 10 previous case reports of spontaneous rupture of pancreatic tumors such as mucinous cyst neoplasms [12, 13], pancreatic neuroendocrine cell tumors [14, 15], solid cystic tumors [16, 17], acinar cell carcinoma [18], pancreatoblastoma [19], and metastasis from other cancers [20, 21]. To the best of our knowledge, no previous English-language report has described spontaneous intra-abdominal bleeding due to rupture of UCP. The reported cases of spontaneous rupture of the pancreas are summarized in Additional file 1: Table S1. Seven patients were female, including two pregnant women, and four patients were male. All patients’ chief complaint was acute-onset upper abdominal pain. Several patients’ general condition rapidly deteriorated after admission. Emergency laparotomies were carried out in all 11 patients. In two patients, internal drainage or cyto-reduction/debulking surgery was chosen because of the patient’s general condition or older age, whereas pancreatectomy was successfully performed in the remaining nine patients. One patient who underwent internal drainage died of severe sepsis originating from a urinary tract infection 3 months postoperatively.

UCP is an extremely rare tumor. In the Pancreatic Cancer Registry in Japan, in which 28,655 cases of pancreatic neoplasms were registered from 1981 to 2004, UCP represents only 0.1% of all pancreatic exocrine neoplasms [22]. The median survival duration of these patients with UCP was 3.3 months, and the 1- and 2-year overall survival rate was 14.4% and 0.0%, respectively. A previous study of 60 cases of UCP showed that UCP tended to present in men (63%) and to be located at the pancreatic head (53%) [23]. The serum concentration of carbohydrate antigen 19-9 (CA19-9) was not elevated in our patient. This is consistent with a previous report in which patients with UCP presented with an elevated serum CA19-9 concentration less frequently than did patients with pancreatic ductal adenocarcinoma [24].

CECT is reportedly essential for prompt diagnosis of intra-abdominal bleeding [6]. We also diagnosed intra-abdominal bleeding due to a pancreatic tumor before surgery based on the CECT findings in this case, but UCP was difficult to diagnose before surgery. According to a previous review of UCP, tumor or rim enhancement on CECT was observed in 82% of cases, and a cyst-like structure was found in the lesion in 47% of cases. The preoperative differential diagnoses were a cystic neoplasm (including intraductal papillary neoplasm, mucinous cystic neoplasm, or serous cystic neoplasm), neuroendocrine neoplasm, acinar cell carcinoma, or solid-pseudopapillary neoplasm, which are occasionally accompanied by hemorrhage, necrosis, and subsequent cystic degeneration within the lesion [23].

In the resected specimens of our case, the tumor was rubbery and fleshy and exhibited cyst formation, hemorrhage, extensive necrosis, and gross infiltration into the adipose tissue. These findings are consistent with the above-mentioned report of a large series of UCP and were presumably due to rapid tumor growth, intra-tumoral hemorrhage, necrosis, and subsequent cystic degeneration [23].

Because of the extremely low incidence of UCP, survival data are controversial and the survival benefit of surgery for UCP remains uncertain. No therapeutic strategies for UCP have been established. A previous report of 35 patients with UCP demonstrated that the 1-, 2-, and 5-year overall survival rates were 59.1%, 30.7%, and 12.2%, respectively, which are comparable with those of pancreatic ductal adenocarcinoma [2]. These results suggest that the survival benefit of radical resection of UCP is similar to that of pancreatic ductal adenocarcinoma. The pathological findings in our case revealed UCP with osteoclast-like giant cells, which was initially described in 1968 by Rosai [25] as a variant of undifferentiated carcinoma. UCP with osteoclast-like giant cells is less aggressive and presumably has a better prognosis than invasive ductal adenocarcinoma of the pancreas [24]. Surgical resection is reasonable when it is possible. Although there is such pathological type associated with hemorrhage as choriocarcinoma with early vascular invasion [26], intra-tumoral hemorrhage does not frequently occur to UCP with osteoclast-like giant cells. In our case, intra-abdominal hemorrhage was caused by the rupture of the distension tumor with intra-tumoral hemorrhage due to the invasion of the tumor cells to splenic vein under anticoagulation therapy.

We have herein reported an extremely rare case of spontaneous rupture of an undifferentiated UCP presenting as intra-abdominal bleeding. Obtaining a correct preoperative diagnosis was quite difficult at the first evaluation. As indicated by our case, UCP with osteoclast-like giant cells should be considered as a differential diagnosis of pancreatic tumors.

## Supplementary Information

**Additional file 1: Table S1.** The reported cases of spontaneous rupture of the pancreas.

## Data Availability

Not applicable.

## Abbreviations

CA19-9: Carbohydrate antigen 19-9
CD68: Cluster of differentiation 68
CECT: Contrast-enhanced computed tomography
UCP: Undifferentiated carcinoma of the pancreas
