# Supplementary material for: Spontaneous rupture of an undifferentiated carcinoma with osteoclast-like giant cells of the pancreas presenting as intra-abdominal bleeding: a case report
Source: Surg Case Rep. 2022 Apr 29;8:79. doi: 10.1186/s40792-022-01437-2 (PMC9051011; doi:10.1186/s40792-022-01437-2)
Supplement: Supplementary file 1 — Additional file 1: Table S1. The reported cases of spontaneous rupture of the pancreas. [file 40792_2022_1437_MOESM1_ESM.docx]

Supplementary Table 1. The reported cases of spontaneous rupture of the pancreas

| **Year** | **Author** | **Age** | **Gender** | **Chief complain** | **Surgical procedure** | **Postoperative course** | **Follow up** | **Pathological diagnosis** |
| --- | --- | --- | --- | --- | --- | --- | --- | --- |
| 1994 | Heim | 85 | F | Abdominal pain | Internal drainage | Dead | 3 months | Cystadenoma |
| 2001 | Ideguchi | 44 | M | Abdominal pain | Distal pancreatectomy | Alive | 77 months | Neuroendocrine carcinoma |
| 2004 | Kobayashi | 53 | F | Abdominal pain | Total pancreatectomy | Alive | 20 months | Metastasis from renal cell carcinoma |
| 2005 | Omori | 31 | F | Abdominal pain | Distal pancreatectomy | Alive | 36 months | Solid cystic tumor |
| 2011 | Nagamura | 32 | F(P) | Abdominal pain | Pancreaticoduodenectomy | Alive | 36 months | Mucinous cystic neoplasm |
| 2012 | Mohammadi | 39 | M | Abdominal pain | Cyto-reduction/ debulking | Alive | 6 months | Acinar cell carcinoma |
| 2013 | Huang | 29 | F(P) | Abdominal pain | Distal pancreatectomy | Alive | 8 months | Solid pseudopapillary neoplasm |
| 2013 | Honda | 5 | M | Abdominal pain | Distal pancreatectomy | Alive | 14 months | Pancreatoblastoma |
| 2020 | Achilli | 32 | F | Abdominal pain | Distal pancreatectomy | Alive | 10 days | Neuroendocrine tumor |
| 2020 | Yokose | 27 | F | Abdominal pain | Distal pancreatectomy | Alive | 6 months | Metastasis from synovial sarcoma |
|  | Our case | 68 | M | Abdominal pain | Distal pancreatectomy | Alive | 3 months | Undifferentiated carcinoma |

(P); pregnant
